# The association between multimorbidity in families and children’s healthcare use: a cross-sectional study of households in the Lolland Falster Health Study (LOFUS)

**DOI:** 10.1186/s12889-026-28419-5

**Published:** 2026-07-10

**Authors:** Louise Bidstrup Jørgensen, Elisabeth Søndergaard, Volkert Siersma, Dagny Ros Nicolaisdottir, Randi Jepsen, Anne Møller

**Affiliations:** 1 The Research Unit for General Practice in Køge, Slagelse, and Copenhagen, and Section of General Practice, Department of Public Health, University of Copenhagen, Copenhagen, Denmark; 2 Lolland-Falster Health Study, Centre for Health Research, Zealand University Hospital, Nykøbing Falster, Denmark

**Keywords:** Household multimorbidity, Child health, Healthcare use, General practice

## Abstract

**Background:**

Chronic illness among adults is common, and an increasing number of children grow up in households where one or more adults live with long-term health conditions. While previous research has shown that parental physical or mental illness may influence children’s mental health, less is known about how adult multimorbidity affects children’s physical health and their healthcare use. This study aimed to investigate the association between household-level multimorbidity and healthcare use among school-aged children.

**Methods:**

This cross-sectional study used data from the Danish Lolland-Falster Health Study (LOFUS), including 1,065 children aged 6–12 years living in 777 households. Children’s questionnaire data were linked to adult data and national health registers. Adult chronic conditions were identified using questionnaire data and ICD-10 diagnoses, and household multimorbidity was defined as ≥ 2 chronic conditions among co-resident adults. Children’s healthcare use, including general practice contacts, out-of-hours/emergency room visits, hospital admissions, and redeemed antibiotic prescriptions, was assessed over 12 months. Associations were analyzed using two-part regression models adjusted for children- and household-level covariates. Analyses were further stratified according to the presence of adult mental illness in the household.

**Results:**

Children living in households with multimorbidity had a significantly higher overall volume of contacts with general practice compared with children from households without multimorbidity. This association was primarily driven by households in which multimorbidity included adult mental illness. Among children who had experienced hospital admission, those from households with multimorbidity had more frequent admissions, although the overall likelihood of admission did not differ between groups. No statistically significant differences were observed for out-of-hours contacts, emergency room visits, or antibiotic prescriptions.

**Conclusions:**

Household multimorbidity was associated with increased healthcare use among children, particularly in relation to general practice. The findings suggest that children’s healthcare use is influenced not only by their own health needs but also by the household’s broader health context. A household-level perspective may therefore contribute to a more nuanced understanding of patterns of healthcare use among children and support clinical awareness in primary care.

## Introduction

In Denmark, many children grow up in families where adults suffer from one or more chronic conditions [1], a trend mirrored globally as increasing numbers of working-age adults live with chronic illness [2, 3]. Estimates suggest that 4–12% of all Danish children and adolescents have a parent with a chronic illness [4, 5].

The family constitutes a central context for child development, where parental health, well-being, and health-related behaviors shape children’s health and well-being [6–11]. Research consistently shows that children of parents with chronic physical or mental illness face an elevated risk of mental health outcomes compared to peers whose parents are healthy [12–16]. However, despite this substantial body of research, far less is known about how parental chronic illness influences children’s physical health. Moreover, most existing studies focus on adolescents, thereby limiting our understanding of how school-aged children are affected by parental illness.

In a previous study of children aged 6–12 years based on household-level data, we found that family conflicts were associated with children’s health complaints, including abdominal pain, back pain, and sleep difficulties, with variations according to socioeconomic status and multimorbidity [17]. These findings raised the question of whether other family-level stressors, such as chronic illness among adults in the household, might similarly be associated with children’s health. While the study focused on self-reported outcomes, the present study builds on the same household-level conceptual framework by examining register-based healthcare use as more objective indicators of children’s health.

Living with a single chronic illness can be demanding, and managing multiple conditions (multimorbidity) often amplifies this burden. When mental illness is part of multimorbidity, challenges typically intensify, increasing strain on family functioning. While multimorbidity primarily affects adults, its consequences may extend to other household members, including children [12–16]. Most previous studies have examined chronic illness in one parent, whereas less is known about how the overall household-level disease burden arising from chronic conditions across multiple adults may be associated with children’s health [12–16]. Given that the burden of multimorbidity primarily affects ill adults but may have indirect consequences for the wider household, this study extends the multimorbidity perspective to the household level. Adult household multimorbidity is defined as the presence of two or more chronic illnesses among adults living in the same household [17]. This perspective reflects the child’s everyday health context and shared household environment, where illness burden may be distributed across multiple adults. Therefore, the aim of this study was to examine whether there is an association between adult household multimorbidity and children’s healthcare use, with particular attention to differences when adult mental illness is part of the household multimorbidity. We hypothesized that children living in households affected by adult multimorbidity have a higher overall use of healthcare services compared with children from households without multimorbidity. We further hypothesized that this association would be stronger in households where multimorbidity included at least one mental illness.

## Material and method

### Study population and procedure

This study has a cross-sectional design and is based on a subsample from the Lolland-Falster Health Study (LOFUS), a household-based health survey including 18,949 participants aged 0–96 years from the two municipalities of Lolland and Guldborgsund in southeast Denmark. Enrollment took place between February 2016 and February 2020 [18]. Adults (≥ 18 years) were randomly selected through the Danish Civil Registration System [19] and invited to participate. Simultaneously, all co-residents registered at the same address were also invited. As participation was voluntary, some households contributed with only one member, whereas others included all household members [17, 20]. Data collection comprised online age-specific questionnaires, clinical examinations, and biological samples [21].

For the present analysis, we used questionnaire data from LOFUS4 (ages 4–10 years), LOFUS11 (ages 11–17 years), and LOFUS18 (≥ 18 years). LOFUS4 was completed by parents or other adults in the household, while LOFUS11 and LOFUS18 were self-administered. Detailed descriptions of the LOFUS study design and procedures have been published elsewhere [20]. The study population for this analysis comprised 1,065 children aged 6–12 years who completed either LOFUS4 or LOFUS11 between February 2016 and February 2018. Only children living in households where at least one adult (≥ 18 years) completed the LOFUS18 questionnaire were included.

The LOFUS data were uploaded to Statistics Denmark and linked with sociodemographic information from administrative registers using the unique Danish personal identification number (CPR), which enables individual-level linkage across national registers and other datasets [19]. Through this linkage, we identified all household members of the included children at the time of invitation to LOFUS, including adults who did not complete LOFUS18.

### Multimorbidity in the household

In this study, a household is defined as all individuals living at the same registered address. We do not assess biological or legal relationships between household members. Instead, we assume that the adults residing with the child constitute the everyday environment in which the child lives and are therefore the individuals whose chronic illnesses may influence the child’s health. Although households often align with what is commonly understood as families, our definition is strictly based on residence.

Chronic illnesses among adults in the household were identified using self-reported data from the LOFUS18 questionnaire, supplemented with diagnostic information from the Danish National Patient Registry (NPR) [22]. The NPR contains complete records of hospital contacts for all Danish residents, including International Classification of Diseases 10th revision (ICD-10) diagnostic codes. The selection of chronic conditions was determined by the disease items included in the LOFUS questionnaire, which have been used consistently across Danish population-based studies [23]. Adult mental illness was defined using self-reported depression or anxiety from the LOFUS questionnaire and hospital-recorded mental disorders identified in the Danish National Patient Registry [22] including ICD-10 codes F30-F39 and F40-F42.

Following Søndergaard et al., we applied the concept of *multimorbidity in the household* (MMH) [17]. For each adult, nine predefined chronic conditions (Appendix 1) were identified using LOFUS18 and NPR data. Households in which the adults collectively had two or more of these conditions were classified as having MMH [17].

### Outcomes

The children’s use of healthcare services was assessed as the number of occurrences across four outcomes: (a) contacts with their general practitioner, (b) contacts with out-of-hours service, defined as contacts with out-of-hours primary care services outside regular GP opening hours or emergency room visits, (c) hospital admissions, and (d) antibiotic prescriptions, defined as the number of redeemed prescriptions for antibiotics. Healthcare use was assessed over a one-year period, including six months before and six months after the completion of the LOFUS questionnaire.

Data on children’s contacts with their general practitioners and out-of-hours service were collected from the National Health Insurance Register [24]. Information on emergency room visits and hospital admissions was obtained from the Danish National Patient Register [22]. Data on redeemed prescriptions for antibiotics were extracted from the National Prescription Registry [25]. Antibiotics were identified using Anatomical Therapeutic Chemical (ATC) codes J01-J05, G01a, and S01a-S02a [26].

### Covariates

The analyses were adjusted for a set of predefined child- and household-level covariates. These included the season of questionnaire completion, child age and sex, household composition (number of children and adults), ethnic background of the household based on adults’ countries of birth, and socioeconomic status defined by employment status. In addition, the presence of chronic illness among the children was included as a covariate. The definition and categorization of covariates have been described in detail elsewhere [17].

### Statistical analysis

The descriptive data of the population of children and the households are shown as numbers with percentages. Differences between households with and without multimorbiditywere examined with chi-squared tests. The associations of MMH with the outcomes listed above were assessed in a two-part model [27]. In the main analysis, households were dichotomized into those with multimorbidity and those without multimorbidity. The first part of the analysis (Part I) assessed the probability of having the outcome using a Poisson regression model, with the differences expressed as risk ratios (RR). This approach allows direct estimation of relative risks rather than odds ratios and was chosen to ensure compatibility with the second part of the model and subsequent calculation of combined effects, following established recommendations for binary outcomes [28]. The second part (Part II) assessed the frequency of the outcome among children who had experienced the event at least once, using Gamma regression, with differences expressed as rate ratios (RaR). Because Part II is restricted to children with observed use, the model estimates relative differences in outcome volume among users rather than differences in overall event counts. The rationale for using this two-part model is that Part I reflects parental healthcare-seeking behavior, whereas Part II reflects the healthcare system’s response and follow-up. A combined effect was obtained by multiplying the RR from Part I, estimated among all children in the study, with the RaR from Part II, estimated among children with at least one observed event, thereby representing the overall difference in outcome volume between the groups. All models were adjusted for the covariates listed above, which were included simultaneously in the models, and clustering of children within households was accounted for using generalized estimating equations (GEE). Models were specified a priori based on theoretical considerations for confounding control rather than model optimization, as the aim was inferential rather than predictive. Accordingly, only fully adjusted models are presented. Finally, we stratified households with multimorbidity according to the presence of mental illness among adults and examined the healthcare use outcomes, also using the above two-part models.

## Results

Characteristics of children and households according to the presence of household multimorbidity are presented in Table 1.

**Table 1:** Characteristics of the study population: 1065 children aged 6–12 years in 777 households

	No MMH^1^	MMH	Total (children)	
	(*n* = 575) *n* (%)	(*n* = 490) *n* (%)	(*n* = 1065) *n* (%)	*p*-value^2^
**Sex**				
Girl	289 (50.2)	236 (48.2)	525 (49.3)	0.497
Boy	286 (49.7)	254 (51.8)	540 (50.7)	
**Age**				
6 years	76 (13.2)	56 (11.4)	132 (12.4)	0.101
7 years	77 (13.4)	58 (11.8)	135 (12.7)	
8 years	91 (15.8)	56 (11.4)	147 (13.8)	
9 years	78 (13.4)	87 (17.8)	165 (15.5)	
10 years	81 (14.0)	74 (15.1)	155 (14.5)	
11 years	85 (14.8)	79 (16.1)	164 (15.4)	
12 years	87 (15.1)	80 (16.3)	167 (15.7)	
**LOFUS questionnaire**				
4–10 years	403 (70.0)	332 (67.8)	735 (69.0)	0.436
11–17 years	172 (30.0)	158 (32.2)	330 (31.0)	
**Chronic illness among children (> 3 mo.)** ^3^				
Yes	41 (7.1)	81 (16.5)	122 (11.5)	< 0.0001
No	534 (92.9)	409 (83.5)	943 (88.5)	
**Season** ^4^				
Winter (Dec, Jan, Feb)	166 (28.9)	122 (24.9)	288 (27.0)	0.207
Spring (Mar, Apr, May)	112 (19.5)	94 (19.2)	206 (19.3)	
Summer (Jun, Jul, Aug)	135 (23.5)	123 (25.1)	258 (24.2)	
Fall (Sep, Oct, Nov)	162 (28.2)	151 (30.8)	313 (29.5)	
	No MMH	MMH	Total (Household)	
	(*n* = 411) n (%)	(*n* = 366) n (%)	(*n* = 777) n (%)	*p*-value^1^
**Socioeconomic status**				
High	337 (82.0)	241 (65.8)	578 (74.4)	< 0.0001
Low	74 (18.0)	125 (34.2)	199 (25.6)	
**Municipality**				
Guldborgssund	270 (65.7)	221 (60.4)	491 (63.2)	0.139
Lolland	141 (34.3)	145 (39.6)	286 (37.8)	
**Ethnic household background** ^5^				
All adults are Danish	284 (69.1)	343 (93.7)	727 (93.6)	0.498
Mixed household	13 (3.2)	17 (4.6)	30 (3.9)	
All adults are non-Danish	14 (3.4)	6 (1.6)	20 (2.6)	
**Number of children (< 18 years) in the household**				
1 child	82 (20.0)	80 (21.9)	162 (20.9)	0.536
2 + children	329 (80.0)	286 (78.1)	615 (79.2)	
**Number of adults (> 18) in the household**				
1 adult	50 (12.2)	24 (6.6)	74 (9.5)	0.027
2 adults	333 (81.0)	318 (86.9)	651 (83.8)	
+ 3 adults	28 (6.8)	24 (6.6)	52 (6.7)	

^1^Multimorbidity in the household (MMH)

^2^*P*-value of a Monte Carlo chi-squared test for the difference in distribution of the covariate between (children from) households with and without MM

^3^ This characteristic was not defined in Søndergaard 2023. Here, it was defined as ‘Yes’ when long-lasting (>3 months) illness or handicap was indicated at the time of the LOFUS questionnaire.

^4^ Season: time of year at questionnaire completion

^5^ Defined from the birthplace of the adults in the household

This study is based on 1065 children aged 6–12 years from 777 households. Households with multimorbidity had lower socioeconomic status and a higher prevalence of chronic disease among the children. They were also more likely to comprise two adults. Given the definition of multimorbidity in the household, two-adult households are, by construction, more likely to qualify because conditions are summed across adults. In contrast, a single-adult household would require more than two conditions in the same adult.

Based on the characteristics, differences in children’s healthcare use according to household multimorbidity are examined in Table 2, which presents results from a two-part model assessing prevalence of use, volume of use among users, and the overall volume of use across healthcare services.

**Table 2:** Differences in children’s healthcare use according to household multimorbidity results from a two-part model

Total (*n*)			With outcome *n* (%)	Part l (95% CI)	*p*-value	Mean (SD)	Part ll (95% CI)	*p*-value	Combined effect (95% CI)	*p*-value
				Prevalence of use			Frequency of use for those who used the service			
**General practitioner**										
No MMH^1^		473	235 (49.7)	(ref)		1.96 (1.65)	(ref)		(ref)	
MMH		395	227 (57.5)	1.14 (1.00;1.29)	0.038	2.14 (1.76)	1.16 (1.00;1.35)	0.055	1.32 (1.09;1.61)	0.005
**Antibiotic prescription**										
No MMH		575	77 (13.4)	(ref)		1.43 (0.82)	(ref)		(ref)	
MMH		490	69 (14.1)	0.98 (0.71;1.33)	0.880	1.64 (1.27)	1.11 (0.90;1.36)	0.344	1.08 (0.74;1.57)	0.690
**Hospital admission**										
No MMH		473	19 (4.0)	(ref)		1.21 (0.54)	(ref)		(ref)	
MMH		395	21 (5.3)	1.10 (0.60;2.03)	0.771	1.38 (0.80)	1.60 (1.15;2.21)	0.005	1.75 (0.87;3.53)	0.120
**Out-of-hours services and emergency room visits**										
No MMH		473	89 (18.9)	(ref)		1.70 (1.00)			(ref)	
MMH		395	87 (22.0)	1.14 (0.86;1.51)	0.343	1.90 (1.41)	1.18 (0.98;1.41)	0.076	1.35 (0.97;1.88)	0.078

^1^*MMH* Multimorbidity in the household

### Prevalence of use (Part I)

Children from households with multimorbidity were in contact with their GP significantly more often than children from households without MMH, with a 14% higher likelihood of GP contact (RR 1.14, 95% CI 1.00-1.29). This reflects a higher probability of having at least one GP contact during the observation period. For the three other outcomes, no statistically significant differences in healthcare use were observed between children from households with or without multimorbidity.

### Volume of use among users (Part II)

In the Part II analysis, children from households with multimorbidity who had been in contact with their GP tended to have a higher volume of GP use than children from households without multimorbidity (*p* = 0.055). This indicates that among children who had at least one GP contact, the volume of use was higher, although not statistically significant. In contrast to the results in Part I analysis, children from households with multimorbidity, who had been admitted to hospital, had a 60% higher volume of hospital admissions (RaR 1.60, 95% CI 1.15–2.21). This means that among children who had experienced at least one hospital admission, those from households with multimorbidity were admitted more often.

### Combined effect

We observed no significant differences in overall antibiotic prescription patterns between the two groups (*p* = 0.690). Similarly, the overall hospital admission volume of use did not differ significantly between the two groups (*p* = 0.120). For GP contacts, the combined effect indicated a significantly higher overall volume of use (32%) of contact among children from households with MMH compared with those from households without multimorbidity (95% CI 1.09–1.61, *p* = 0.005) For the use of emergency services, comprising out-of-hours consultations and emergency room visits, no statistically significant differences were found, although children from households with MMH showed a 35% higher overall volume of use (95% CI 0.97–1.88, *p* = 0.078).

To assess whether these associations differed by the presence of adult mental illness in the household, analyses were stratified accordingly. Results are presented in Table 3.

**Table 3:** Children’s healthcare use according to household multimorbidity: prevalence, frequency, and combined effect

Total *n*		With outcome *n* (%)	Part l (95% CI)	*P*-value	Mean (SD)	Part ll (95% CI)	*P*-value	Combined effect (95% CI)	*P*-value
			Prevalence of use			Frequency of use for those who used the service			
**General practitioner**									
No MMH	473	235 (49.7)	(ref)		1.96 (1.65)				
MMH^1^	294	172 (58.5)	1.12 (0.98;1.28)	0.097	2.04 (1.80)	1.10 (0.93;1.30)	0.290	1.23 (0.99;1.53)	0.062
MMHm^2^	101	55 (54.5)	1.07 (0.86;1.32)	0.550	2.48 (1.60)	1.46 (1.l8;1.80)	< 0.001	1.55 (1.15;2.10)	0.004
**Antibiotic prescription**									
No MMH	575	77 (13.4)	(ref)		1.42 (0.82)				
MMH	356	50 (14.0)	0.94 (0.67;1.33)	0.740	1.7 (1.38)	1.12 (0.89;1.41)	0.330	1.06 (0.70;1.59)	0.790
MMHm	134	19 (14.2)	0.99 (0.60;1.63)	0.970	1.47 (0.96)	1.07 (0.77;1.49)	0.690	1.06 (0.58;1.92)	0.850
**Hospital admission**									
No MMH	473	19 (4.0)	(ref)		1.21 (0.54)				
MMH	294	14 (4.8)	0.95 (0.49;1.84)	0.880	1.42 (0.94)	1.93 (1.30;2.86)	0.001	1.82 (0.84;3.97)	0.130
MMHm	101	7 (6.9)	1.50 (0.63;3.56)	0.360	1.29 (0.49)	1.78 (1.26;2.50)	< 0.001	2.66 (1.05;6.76)	0.039
**Out-of-hours services and emergency room visits**									
No MMH	473	89 (18.8)	(ref)		1.71 (0.99)				
MMH	294	66 (22.4)	1.11 (0.83;1.50)	0.490	1.98 (1.49)	1.15 (0.94;1.41)	0.180	1.28 (0.89;1.83)	0.180
MMHm	101	21 (20.8)	1.15 (0.72;1.84)	0.550	1.62 (1.11)	1.13 (0.87;1.46)	0.350	1.30 (0.77;2.21)	0.330

*MMH *Multimorbidity in the household

*MMHm *Multimorbidity in the household with the presence of mental illness

### Stratified analysis

When stratifying for the presence of mental illness as one of the chronic diseases in the household, we found that the significantly higher overall volume of GP use was primarily driven by children from households where at least one chronic condition was a mental illness (Table 3). In the combined analysis, these children had a 55% higher overall volume of GP use, compared to children from households without multimorbidity (95% CI 1.15–2.10, *p* = 0.004).

Compared with children from households without multimorbidity, the volume of hospital admissions among users in Part II analysis was higher for children from households with multimorbidity, both with and without mental illness (*p* = 0.001 and p = < 0.001, respectively). In contrast to the former analyses, the probability of having at least one hospital admission (Part I) was higher and statistically significant, only for children from households in which at least one chronic condition was a mental illness (95% CI 0.63–3.56, *p* = 0.036).

No statistically significant differences were observed across the three groups for out-of-hours contacts, emergency room visits, or antibiotic prescriptions.

## Discussion

This study indicates that household multimorbidity is associated with an increase in children’s healthcare use. Compared to children from households without multimorbidity, those living in households with multimorbidity had a higher likelihood of GP contact, a tendency toward more frequent GP contacts among children with at least one GP contact, and a significantly greater overall volume of GP contacts. The likelihood of hospital admission did not differ between groups; however, among admitted children, those from households with multimorbidity experienced more admissions. These differences were most pronounced in households where multimorbidity included adult mental illness. Rather than focusing on illness in a single identified parent, this study considers multimorbidity at the household level, reflecting the overall burden of chronic illness among adults living with the child. Such an approach acknowledges that children’s exposure to illness may arise from the wider household context rather than from one individual adult alone.

### Potential mechanism and healthcare-seeking behavior

Several factors may help explain these patterns. The child’s experience and reporting of symptoms is the first step in any healthcare contact. Parents’ or caregivers’ interpretations of these symptoms, along with their usual healthcare-seeking behavior, influence whether and when care is sought. Previous experiences with the healthcare system and the family’s familiarity with healthcare professionals may influence parents’ decisions to seek care and communicate concerns, as well as subsequent clinical decision-making made within the healthcare system. These mechanisms align with research suggesting that family norms and responses to illness can influence healthcare use across household members [6, 29].

Research indicates that families share not only physical environments but also norms, beliefs, and patterns of behavior that shape responses to symptoms and interactions with healthcare services [6]. Parents and other caregivers act as primary models for interpreting and managing illness, and children may adopt these patterns over time [6]. In households affected by chronic illness, repeated contact with healthcare services may reinforce a norm in which seeking professional care becomes a familiar and expected response.

### Differences across healthcare services

Within this context, patterns of GP contact illustrate how the overall health situation in the household may shape decisions about seeking care for children. As described in previous research, decisions to seek healthcare are influenced not only by the child’s immediate symptoms but also by parents’ experiences, expectations, and familiarity with the healthcare system [6, 30, 31]. In this study, multimorbidity was associated with higher GP use among children, even after adjustment for socioeconomic status. This finding aligns with previous research showing that parental illness is linked to higher healthcare use among children [6, 30, 32]. Adults living with chronic illness often have repeated contact with healthcare services, which may influence how readily care is sought for children in the same household [29]. In addition to family-initiated healthcare seeking, increased primary care use may also reflect GP-initiated contacts. GPs may proactively schedule follow-up visits or monitor children’s well-being more closely in households characterized by a high burden of chronic illness. However, the register data does not indicate whether contacts were initiated by the family or by the GP, and we were therefore unable to examine this further.

Differences in the types of outcomes examined in this study may reflect different underlying processes. GP contacts, antibiotic prescriptions, and out-of-hours contacts are likely to be more closely related to healthcare-seeking initiated by parents or caregivers. In contrast, hospital admissions are more dependent on clinical assessment and decisions made within the healthcare system. This distinction may help explain why patterns of use varied across outcomes. Hospital admissions may therefore be less sensitive to household-level health behaviors and more reflective of clinical severity. For antibiotic prescriptions and out-of-hours contacts, no apparent differences were observed between children from households with and without multimorbidity, suggesting that the association may not be uniform across all types of healthcare services.

### Role of adult mental illness in the household

The stratified analyses showed that the association between household multimorbidity and children’s healthcare use was most apparent when mental illness was present among adults in the household. This supports our hypothesis that adult mental illness is an important contextual factor, as it can affect family functioning and caregiving capacity, influencing healthcare-seeking for the child. Mental illness may also affect parental stress levels, perceived coping capacity, and thresholds for seeking care, which could amplify healthcare use among children. Previous research has linked parental mental illness to increased healthcare needs and vulnerabilities among children [33]. By focusing on school-aged children within a household framework, our findings add insight into how family health contexts shape healthcare use in this age group. Taken together, these findings indicate that household multimorbidity, particularly when adult mental illness is present, constitutes an important contextual factor for understanding variation in children’s healthcare use, rather than a direct marker of child morbidity or treatment need.

### Strengths and limitations

A key strength of this study is the use of data from the Lolland-Falster Health Study, which includes information on all household members. This enables a household-level analysis of multimorbidity in a population-based setting and provides a broader view of the environment in which children live. The study also distinguishes between households with and without adult mental illness, allowing for a more nuanced assessment. The selection of chronic conditions was limited to disease items included in the LOFUS questionnaire and used in previous large population-based studies. This ensures standardized assessment but does not capture all possible chronic conditions. The regional context is important to consider. Lolland-Falster is characterized by higher socioeconomic deprivation than other areas of Denmark [21, 34, 35], which is often associated with poorer health and may influence healthcare patterns independently of SES adjustments. As described above, this contextual setting may influence baseline levels of healthcare contact among children and thereby the observed differences between household groups. Although a non-participation analysis showed no major differences between LOFUS participants and non-participants [35] the generally higher healthcare needs in this region may partly explain the lack of difference in GP contact between children from households with multimorbidity without mental illness and those from households without multimorbidity. Further, household composition was defined at the time of participation, and changes such as joint custody or residential moves during the one-year outcome period may have led to some misclassification, which could have attenuated associations. Household composition also differed between groups, with a higher proportion of single-adult households among children living in households without multimorbidity. Although analyses were adjusted for the number of adults and children in the household, residual influences of family structure on healthcare use cannot be fully excluded.

Additional limitations include potential underrepresentation of mental illness, as diagnoses made solely in general practice were not captured. Defining multimorbidity at the household level means that we did not specify which adult was affected, their caregiving roles, or differences between maternal, paternal, or other caregiver illness. Furthermore, some chronic conditions may not have been recorded in the available data sources, potentially reducing contrasts between groups. Finally, as a cross-sectional analysis, the study cannot establish causation or clarify how physical and mental illness within households interact over time.

## Conclusion

This study demonstrates that household multimorbidity is associated with increased healthcare use among school-aged children living in Lolland-Falster, particularly in relation to GP services. Children living in households with multimorbidity had both a higher likelihood of GP contact, more frequent GP contact among users, and a higher overall volume of GP contact compared with children from households without multimorbidity. Hospital admission rates did not differ between groups. However, among children who were admitted, those from households with multimorbidity experienced more admissions. These associations were most pronounced when adult mental illness was part of the household multimorbidity profile.

The findings suggest that children’s healthcare use is shaped not only by their own health needs but also by the broader health context of the household. Adults’ illness experiences, healthcare-seeking patterns, and repeated contact with healthcare services may influence decisions about seeking care for children. Mental illness appears to be a particularly important factor, potentially affecting family functioning and caregiving capacity.

By adopting a household-level perspective, this study adds new insight into how family health contexts relate to healthcare use among school-aged children. Future research should explore these dynamics longitudinally and examine how different caregiving roles and household structures contribute to patterns of healthcare utilization. Understanding these mechanisms may inform clinical awareness and future interventions aimed at supporting families affected by multimorbidity and mitigating potential consequences for children’s health.

## Data Availability

The data supporting this study’s findings were provided by LOFUS and Statistics Denmark. Restrictions apply to these data, which were used under license for the current study and are therefore not publicly available. Data are, however, available from the authors upon reasonable request and with permission of LOFUS and Statistics Denmark.

## Appendix 1

**Table:**

Original response categories in LOFUS18	Grouping into the eight largest disease groups in DK + ’other’	Corresponding ICD-10 mapping
Coronary thrombosis	Cardiovascular disease	I20-I25, I70-I79, I80-I82
Atherosclerosis of the heart		
Cardiac spasm (angina pectoris)		
Deep vein thrombosis		
Diabetes	Diabetes	E10-E14
Asthma	Hypersensitivity diseases and COPD	J30, J40-J47
Allergy (not eczema)		
Chronic bronchitis, emphysema, smoker’s lungs, Chronic Obstructive Pulmonary Disease (COPD)		
Kidney disease	Kidney disease	N00-N08, N10-N16, N17-N19
Anxiety	Mental disorders	F30-39, F40-42
Depression		
Cancer	Cancer	C00-C96
Osteoarthritis	Musculoskeletal disorders	M05, M06, M15-M19, M50-M54
Arthritis		
Herniated disc or other back disorders		
Migraine or frequent headache	Headache	G43
Other	Other	B18, B21-B24, B150, B160, B162, B190, D86, F00-F03, F051, G30, G45, G46, G81, G82, I12, I13, I50, I60-I69, I85, I110, I130, I132, J60-J67, J684, J701, J703, J841, J920, J961, J982, K25-K28, K71-K74, K221, K700-K704, K709, K760, K766, K983, M08, M09, M30-M36, Q61

^a^ The ICD-10 codes included in Other are part of Charlson's Comorbidity Index (CCI) (Charlson et al., 1987) and do not appear in any of the other eight disease groups. CCI is an established method of categorising comorbidities of patients based on the International Classification of Diseases (ICD-10)

^b^ Because hypertension is classified as a risk factor, not a disease, we removed it from the list

## Abbreviations

MMH: Multimorbidity in the Household
LOFUS: Lolland-Falster Health Study
GP: General Practitioner
NPR: Danish National Patient Registry
ICD-10: International Classification of Diseases, 10th revision
ATC: Anatomical Therapeutic Chemical classification system
RR: Risk Ratio
RaR: Rate Ratio
GEE: Generalized Estimating Equations
SD: Standard Deviation
CI: Confidence Interval
SES: Socioeconomic Status
